# Origine de la syphilis en Europe : fin d'une controverse ?

**DOI:** 10.48327/mtsi.v5i1.2025.666

**Published:** 2025-03-21

**Authors:** Jean-Paul LOUIS, Francis LOUIS

**Affiliations:** diplômé de médecine tropicale, titulaire du CES de santé publique, spécialiste de recherches du Service de santé des armées en lutte contre les grandes endémies; diplômé de médecine tropicale, spécialiste des hôpitaux des armées en biologie et diplômé de santé

**Keywords:** Syphilis, Tréponématoses, Amériques, Europe, Études phylogénétiques, Syphilis, Treponematoses, Americas, Europe, Phylogenetic Studies

## Abstract

Au retour de Christophe Colomb, une maladie inconnue est découverte à Barcelone en Espagne en 1493, avant d'apparaître à Naples en Italie en 1494/1495 lors d'une guerre avec la France. D'abord décrite dans les troupes, elle a rapidement diffusé dans toute l’Europe quand les armées se sont retirées. La question qui se pose est de savoir s'il y a une relation de cause à effet avec le retour de Christophe Colomb en Espagne, ou s'il s'agit d'une simple coïncidence car la syphilis semble avoir été présente en Europe avant l'arrivée des Européens en Amérique, mais elle n'aurait pas été identifiée comme telle. Ceci expliquerait qu'elle n'ait pas été clairement perçue par la population ni décrite dans la littérature disponible. Récemment, des recherches archéologiques et paléopathologiques sur des restes humains de l’époque moderne, appuyées par les données de la génétique, ont clairement établi la présence de la syphilis en Europe dans les temps anciens. Ces données montrent également que la syphilis pourrait ne pas avoir existé dans les Amériques à l’époque colombienne. Il semble cependant possible que les compagnons de Christophe Colomb aient rapporté en Europe une souche non vénérienne de tréponématose qui aurait muté lors de son adaptation aux conditions environnementales nouvelles, augmentant sa pathogénicité et modifiant son mode de transmission lors du transfert sur de nouveaux sujets, peut-être les prostituées. En retour, cette souche de syphilis vénérienne aurait contaminé le continent américain lors des conquêtes espagnoles et/ou la traite des esclaves noirs. Cette étude apporte quelques arguments récents pour nourrir la controverse.

## Abstract (en)

Upon Christopher Columbus's return, an unknown disease was discovered in Barcelona, Spain, in 1493, before appearing in Naples, Italy, in 1494/1495 during a war with France. Initially described among the troops, it quickly spread throughout Europe as the armies withdrew. The question arises whether there is a cause-and-effect relationship with Columbus's return to Spain or if it is merely a coincidence, as syphilis seems to have been present in Europe before Europeans arrived in the Americas, though it may not have been identified as such. This would explain why it was not clearly recognized by the population or described in the available literature. Recently, archaeological and paleopathological research on human remains from the modern era, supported by genetic data, has clearly established the presence of syphilis in ancient Europe. These findings also suggest that syphilis may not have existed in the Americas during Columbus's time. However, it seems possible that Columbus's companions brought back to Europe a non-venereal strain of treponematosis, which could have mutated while adapting to new environmental conditions, increasing its pathogenicity and altering its mode of transmission when transferred to new individuals, possibly prostitutes. In turn, this venereal syphilis strain may have infected the American continent during the Spanish conquests and/or the transatlantic slave trade. This study provides some recent arguments to fuel the controversy.

La thèse communément admise soutient que la présence de la syphilis en Europe, supposée endémique en Amérique, serait liée à son introduction par Christophe Colomb. Cette origine colombienne rencontre une large unanimité qui perdure si l'on s'en réfère aux récentes publications de spécialistes reconnus : « *À la Renaissance, la syphilis est ramenée par Christophe Colomb d’Amérique où elle sévissait à l’état endémique. Cette maladie jusque-là inconnue et souvent rapidement mortelle n'avait pas été décrite par les médecins grecs réputés infaillibles* » [9].

À l'opposé, les travaux de paléopathologie menés ces dernières décennies s'orientent vers une origine ancienne précolombienne de la syphilis en Europe, ce qui amène certains à être plus circonspects dans leurs affirmations. En témoigne Bueno : « *Tandis que la variole, qui s'est propagée en un temps record dans le Nouveau Monde, est venue d’Europe, il semblerait que la syphilis ait été importée des Amériques* » [11].

Les origines de la syphilis font en effet l'objet de débats passionnés. L'histoire évolutive de cette maladie sexuellement transmissible oppose globalement les partisans de deux thèses : les « américanistes » qui plaident pour l'introduction de la maladie en Europe par les équipages de Christophe Colomb rentrant du Nouveau Monde en 1493, et d'autres qui défendent la présence de l'infection en Europe et dans les autres régions de l’Ancien Monde avant cette date charnière. La vivacité des controverses tient en partie à la complexité du sujet qui suscite de multiples discussions épidémiologiques, historiques, cliniques, bactériologiques, paléopathologiques [16]. C'est finalement tout le débat entre le mémoriel et l'historique en faisant ici référence à Fabiani-Salmon « *Et si l'histoire de la médecine pouvait aussi confondre l'historique et le mémoriel ?* » [17]. Ce débat fait appel à des éléments cliniques, épidémiologiques, bactériologiques et paléopathologiques [16].

Sur le plan clinique, en plus de la syphilis, on dénombre trois autres tréponématoses humaines se caractérisant par leur mode de contamination et leur distribution géographique (pian, béjel et pinta). La syphilis vénérienne est transmise principalement par voie sexuelle chez les adultes mais aussi par voie congénitale et s'observant chez le nouveau-né. Elle prévaut actuellement sur tous les continents. Le pian est une infection des régions tropicales chaudes et humides qui se transmet par contact cutané. Le béjel est une tréponématose des zones désertiques et semi-désertiques se transmettant dès l'enfance par voie orale. Enfin la pinta à transmission cutanée est une tréponématose uniquement dermatologique, qui n'est présente que dans une partie de l’Amérique latine et n'est donc pas concernée par la question.

En dehors de cette dernière, les trois autres tréponématoses ont une expression clinique et une évolution classique mais pas systématique en trois phases :

- Une phase primaire suivant la contamination, marquée par un chancre d'inoculation hautement contagieux dont la localisation dépend du mode de transmission, génitale pour la syphilis, cutanée pour le pian, buccale pour le béjel.
- Une phase secondaire apparaissant en quelques mois, marquée par des signes cutanés d'expression très variable et l'apparition de plaques muqueuses indolores dont la localisation au niveau des paumes de mains et des plantes des pieds est évocatrice.
- Une phase tertiaire dont l’évolution, qui se mesure en années après la contamination, s'exprime au niveau viscéral et cutanéo-muqueux. C'est à ce stade que sont constituées les lésions osseuses caractéristiques intéressant les paléopathologistes, l'ostéopériostite gommeuse avec deux localisations de prédilection : le crâne et les tibias.

Sur le plan bactériologique, la question posée est de savoir s'il existe une ou plusieurs espèces de tréponèmes. Cette question est longtemps restée en suspens faute de pouvoir cultiver les tréponèmes. L'approche génétique permet d'apporter un début de réponse.

L'absence de différences bactériologiques (morphologique, biochimique, sérologique, immunitaire) et la très faible distance génétique entre les souches identifiées chez des patients atteints de syphilis, de pian et de béjel, a amené à supposer qu'une seule et même espèce, *Treponema pallidum*, était responsable d'une seule maladie, les différentes expressions s'expliquant par des modes de contamination variables, eux-mêmes dépendant du climat et des habitudes culturelles [7].

Une conception plus actuelle retient des sous-espèces au sein de l'espèce *Treponema pallidum* spp. : *pallidum* pour la syphilis (TPA), *pertenue* pour le pian (TPE) et *endemicum* pour le béjel (TEN). Les progrès dans le séquençage des génomes ont permis de confirmer ce point de vue taxonomique pour les trois sous-espèces *pallidum, pertenue* et *endemicum.* Ces différences génétiques minimes sont insuffisantes pour les distinguer en tant quespèces. Les importantes différences épidémiologiques et cliniques pourraient être liées à des variations dans les gènes intervenant sur l'infectiosité et la virulence, ce qui pourrait expliquer les changements de mode de transmission, y compris les modes épidémiques et d'expression clinique [36].

Les tréponématoses sont responsables d'une atteinte osseuse au stade tardif de la maladie, mais de façon plus précoce chez le nouveau-né et l'enfant en cas de syphilis congénitale. Cette ostéopériostite touchant électivement le crâne et les os longs est la base des critères paléopathologiques d'identification des tréponématoses sur les restes osseux de squelettes archéologiques. Pour les formes congénitales, uniquement observées en cas de contamination vénérienne, de nombreuses lésions sont évocatrices : ostéochondrite, ostéomyélite, périostite, dactylite, anomalies dentaires. Peu d'auteurs en contestent le bien-fondé en paléopathologie.

Les hypothèses concernant la genèse du développement de la syphilis doivent donc s'apprécier à la lumière de ces différents éléments.

## À l'origine de l'histoire : Christophe Colomb

Au retour de Christophe Colomb, une maladie inconnue aurait été détectée à Barcelone en 1493 avant de se manifester en 1494/1495 en Italie, à Naples, lors d'une invasion française. D'abord déclarée au sein des troupes, elle s'est rapidement répandue dans toute la péninsule italienne, puis en France avec le retour des armées, et enfin dans l’Europe entière. Existe-t-il une relation de cause à effet avec le retour en Espagne de Christophe Colomb ou est-ce une simple coïncidence et quels arguments peut-on apporter pour tenter d'y répondre ?

Disposant d'une caraque, la *Santa Maria* qu'il commande lui-même, et de deux caravelles, la *Niña* et la *Pinta* commandées respectivement par Vicente Yáñez Pinzón et Martin Alonso Pinzón, Christophe Colomb embarque le 3 août 1492 avec 87 matelots, espagnols et d'autres nationalités. Le 12 octobre 1492, il accoste sur l’île de Guanahani aux Bahamas qu'il baptise San Salvador. L'accueil des indigènes Taïnos est très chaleureux et la grande liberté de mœurs des femmes, jointe à l'abstinence forcée des matelots, entraine de nombreux contacts sexuels. Dans son journal de bord (1492-1493), Colomb fait allusion à ces rapports sexuels des équipages, y compris Martin Pinzón, avec les jeunes filles Taïnos. Le 28 octobre, il relâche à Cuba puis aborde Hispaniola (Saint Domingue) le 6 décembre. Dans un rapport au roi d’Espagne en 1526, l'historien Gonzalo Fernandez d’Oviedo relate qu'il y eut à Hispaniola un « commerce » très intense entre les matelots et les femmes des Caraïbes.

La *Santa Maria* ayant coulé, Christophe Colomb doit laisser une partie de l’équipage sur place. Il est de retour des « Indes » avec les 2 caravelles, 44 matelots et 6 indigènes le 4 mars 1493 au Portugal. Il aborde ensuite à Séville le 31 mars, puis il se rend à Barcelone le 7 mai où il présente au roi les indigènes qu'il a ramenés. Il y reste jusqu'au 25 septembre 1493, date de son second voyage avec 17 navires et 1 500 hommes destinés à établir une colonie. Il ramène avec lui les trois amérindiens survivants. Il ne reviendra en Espagne qu'en juin 1496. Notons cependant le retour en Espagne le 12 février 1494, sous le commandement d’Antonio de Torès, de 12 navires ramenant en particulier des malades, sans autre précision, ainsi que 30 esclaves des Caraïbes présentés, dans une lettre d'accompagnement de Colomb à son souverain, comme des « *cannibales, hommes, femmes, garçons et fillettes* » [15].

## La guerre d'Italie et la fin du XV^e^ siècle

En septembre 1494, le roi de France Charles VIII envahit l’Italie à la tête d'une armée de 15 à 20 000 hommes, mercenaires flamands, gascons, suisses, italiens et espagnols, suivis comme de coutume à l’époque, par une cohorte de gueux et de prostituées [33]. Le roi conquiert Florence puis Rome abandonnée par les troupes espagnoles et napolitaines, et enfin Naples le 28 janvier 1495, alors sous la dépendance du roi d’Aragon qui y maintenait une garnison d'un millier d'hommes, espagnols mais aussi italiens et allemands [18]. En mars 1495, une coalition se met en place avec des effectifs nombreux, italiens, espagnols et autrichiens. Charles VIII quitte Naples devant le nombre de ses ennemis qui arrivent, mais aussi du fait de la conduite scandaleuse de ses mercenaires qui, par leur comportement paillard et débauché, se sont attiré l'hostilité populaire. Il se replie sur Fornoue le 5 juillet 1495 où il est battu. À ce stade, les troupes françaises sont déjà touchées par un mal nouveau qui fait l'objet des premières descriptions précises et évocatrices comme celle de Marcellus Cumanus, chirurgien des troupes vénitiennes en 1495, qui raconte ce qu'il a vu : « *Il y avait beaucoup de soldats et de fantassins, chez qui j'ai été témoin de l’ébullition des fluides, qui souffraient de démangeaisons, de plusieurs pustules sur la face et sur tout le corps, et commençant communément par le prépuce, ou à l'extérieur du prépuce, comme un grain de mil ou une châtaigne* » [13]. Benedetto, autre médecin vénitien présent à Fornoue, rapporte que « *Par le contact vénérien, une maladie nouvelle, ou du moins inconnue des médecins qui nous ont précédés, le mal français, s'est glissée de l’Occident jusqu’à nous* » [18].

L’épidémie de syphilis se répand rapidement dans les deux camps. Les mercenaires du roi, contraints à la démobilisation, se dispersent dans toute l’Europe où ils diffusent la maladie.

## Hypothèse colombienne

D'aucuns estiment que les soldats espagnols servant dans les deux camps seraient les premiers agents de la dissémination de la syphilis en Europe, mais cela ne dit pas comment eux-mêmes se seraient contaminés [18]. Il parait difficile d'admettre que le faible contingent revenu d’Hispaniola ait pu réussir à propager une épidémie en si peu de temps, ce d'autant que Colomb ne fait nulle part mention d'une telle maladie chez son équipage et qu'il n'a pas hésité à présenter les Indiens à la cour du roi.

Ruy Diaz de Isla, chirurgien portugais vivant à Barcelone, est le premier à proposer la théorie d'une contamination importée. En 1539, dans son « Traité contre le mal serpentin qu'on appelle vulgairement bubas », il écrit :

*J'ai une grande expérience parce que j'ai soigné des personnes ayant fait partie de la première escadre qui découvrit ce pays et sur laquelle revinrent beaucoup de malades atteints de ce mal, et parce que je traitais à Barcelone des malades atteints de ce mal avant que le roi Charles entrât dans Naples*.

Il fait la première description clinique de ce qui lui sembla être la syphilis à partir de ces « nombreux cas » soignés à Barcelone, mais surtout à partir de ses observations sur Martin Pinzón qui serait donc le premier cas connu répertorié. Sauf que Martin Pinzón, arrivé avec son équipage à Palaos de la Frontera le 15 mars 1493, bien loin donc de Barcelone, y était resté car malade et y décéda rapidement, le 31 du même mois. Il est toutefois possible qu’à cette époque la syphilis ait pu entrainer un décès en quelques mois [19]. Notons aussi que ses deux frères, Vicente et Francisco, qui étaient de ce premier voyage à bord des caravelles, continuèrent de naviguer pendant deux décennies sans que l'on ait mention de quelque pathologie que ce soit. Il est aussi permis de s'interroger sur les « nombreux malades de la première escadre » compte tenu de leur effectif très limité.

En 1503 à Hispaniola, Bartolomé de Las Casas commence la rédaction d'une « Histoire générale des Indes » dans laquelle il écrit :

*Il y avait une chose dans cette île qui fut très pénible pour les Espagnols, la maladie des bubas qu'en Italie on nomme mal français : il est avéré qu'elle vînt de cette île, lors du premier retour en Castille de Christophe Colomb avec les premiers Indiens ou quelques Espagnols avec le mal des bubas* … *J'ai pris soin à plusieurs reprises d'interroger les Indiens de cette île si ce mal existait depuis longtemps et ils m'ont répondu affirmativement. Il y a un fait avéré, c'est que tous les Espagnols qui n'observèrent pas la chasteté furent atteints des bubas et que pas un seul n'y échappa.*

Fra Ramon Pané, qui participa au deuxième voyage de Colomb, rapporte dans sa « Relation de l'histoire ancienne des Indiens », un mythe Taïno dans lequel « *Un homme ayant profité d'une femme pour son plus grand plaisir, chercha rapidement un lieu où se laver, étant rempli de ces plaies que nous dénommons le mal français* » [31].

Dans son « Histoire générale des Indes » (1535), Oviedo, qui était à la Cour lors de la présentation des premiers Indiens par Colomb, écrit :

*Votre majesté peut tenir pour certain que cette maladie vient des Indes où elle est très commune chez les Indiens mais pas si dangereuse dans ces contrées que dans les nôtres. La première fois que cette maladie a été vue en Espagne, ce fut après que l'amiral eut découvert les Indes et revint de ces contrées. Et lorsqu'en 1495 le capitaine de Cordova passa en Italie avec une armée, cette maladie y passa avec quelques-uns de ses Espagnols et c'est la première fois qu'on la vit en Italie.*

À noter cependant que ces troupes arrivèrent en Italie alors que celles de Charles VIII avaient déjà quitté Naples.

Les partisans du modèle colombien s'appuient aussi sur d'autres témoignages historiques de contemporains, parfois directement victimes du mal vénérien comme le chevalier Ulrich von Hutten (1488-1523) qui lui consacre un bref traité en 1521. À noter aussi ceux de Niccolo Leonicensus (1428-1524), Jean de Vigo (vers 1450-1525), Jérôme Fracastor (1478-1553) ou Johannes Baptista Montanus (vers 1489-1551). Tous ces textes relatent la survenue dans tous les pays d’Europe, entre la fin du XV^e^ siècle et le début du XVI^e^ siècle, d'une épidémie jusqu'alors inconnue, frappant les esprits par sa gravité et son apparente nouveauté [16].

## Hypothèse pré colombienne : la syphilis et l'Europe

Cette hypothèse soutient que la syphilis était présente en Europe avant l'arrivée des Européens en Amérique mais sans être identifiée comme telle. Les documents écrits sont rares, le diagnostic de cette maladie étant difficile à poser d'après les descriptions des textes antiques ou médiévaux antérieurs au XV^e^ siècle où, selon la tradition hippocratique, on nommait *lepra* toute manifestation cutanée. Il n'est donc pas surprenant que les chroniqueurs du temps n'aient rien relaté de précis sur une maladie qui n’était pas décrite ni nommée, peut-être aussi moins virulente, donc plus rare, et dont les manifestations cliniques pouvaient prêter à confusion.

Les défenseurs du modèle précolombien mettent cependant en avant des textes antérieurs relatant l'existence en Europe d'un mal vénérien caractérisé par des ulcérations génitales, parfois confondu avec la lèpre quand il s'accompagne d'ulcérations faciales et des membres inférieurs : Bartholomé de Glanville (?- vers 1360) parle d'une lèpre vénérienne et congénitale, Bernard de Gordon (XIII^e^-XIV^e^ siècles), Guy de Chauliac (vers 1300-1368) et Valescus de Tarente (XIV^e^-XV^e^ siècles) citent des ulcérations de la verge survenant après des rapports charnels avec des femmes contaminées. Ces témoignages anciens portant sur des descriptions de la maladie ont pu être taxés de non typiques, non fiables ou dont la date a été mal calculée [32]. Objet de telles controverses, le poème « Sylva in scabiem » (De l'ulcération) d’Ange Politien daté entre 1475 et 1478 pourrait décrire la maladie, ce que ne confirment pas exactement Moulin et Delort [29]. Pour eux, « *Ce cas pose sans le résoudre le problème des origines de la syphilis* ».

La paléopathologie apporte des éléments scientifiques *a priori* plus probants que ces textes discutés. Au départ, il faut prendre en compte les travaux de paléopathologie menés par Hackett et Steinbock en 1976 puis par Resnick en 1988, qui ont montré qu'une étude morphologique détaillée d'un ensemble lésionnel permettait, à partir de stigmates ostéopériostiques, de retenir le diagnostic de tréponématose. Ces travaux ont été complétés par ceux de Rothschild *et al.* en 1995 qui ont proposé une méthode de diagnostic différentiel entre les tréponématoses [34]. Des réserves sont cependant émises, mettant en avant qu'il est difficile sinon impossible de distinguer de façon indiscutable les lésions observées à partir de l'analyse d'un squelette à laquelle des tréponématoses sont attribuables. Les différences s'expriment seulement en fréquence d'atteinte préférentielle des localisations osseuses [7, 8].

Sur ces bases, Brun *et al.* ont fait l'inventaire en 1998 des données archéologiques et paléopathologiques des tréponématoses antérieures au XVI^e^ siècle [10]. Ces données tendent à montrer que la syphilis était présente dès l’Antiquité en Angleterre, en France, en Italie, en Autriche et en Croatie. À noter en particulier le cas du fœtus de Costebelle (Var) daté du IV^e^ siècle qui présente un ensemble exceptionnel de lésions osseuses permettant de poser avec une quasi-certitude le diagnostic de syphilis congénitale précoce. D'autres cas possibles de syphilis congénitale ont également été mis en avant dans d'autres pays européens, des travaux ultérieurs étant venus compléter et préciser ces premières observations [2, 3, 14, 20, 24, 30].

Les cas archéologiques restent encore rares. Mais le développement systématique des recherches autorisé par la rupture du dogme colombien pourrait permettre de compléter nos connaissances sur l'histoire des infections à tréponèmes en Europe. Si des doutes persistent quant à la fiabilité des diagnostics retenus, il convient de prendre en compte l'identification moins discutable des cas de syphilis congénitale.

## Une version portugaise de l'origine de la syphilis

En 1991, Livingstone, frappé par la concordance temporelle et géographique entre la fréquentation des côtes africaines et la survenue de l’épidémie de syphilis, a soutenu que cette épidémie qui aurait déferlé sur l’Europe aurait été apportée dans la péninsule ibérique par les marins portugais [1]. Les Portugais ont abordé le Cap Vert dès 1444, la Côte de l’Or à partir de 1460, l'embouchure du Congo en 1483 et le cap de Bonne Espérance en 1487. C'est ainsi qu'en 1444, une expédition est revenue au Portugal avec une première cargaison de 235 esclaves et que, pour faire face au développement de la traite négrière, la Maison des esclaves a été fondée à Lisbonne en 1486. Des cas de pian auraient pu être ainsi amenés et leurs caractéristiques modifiées par la transposition dans des conditions culturelles et climatiques différentes. Un foyer épidémique aurait pu se former entre 1460 et 1490, l’épidémie couver à bas bruit et faire explosion à la fin du siècle au moment où le retour de l'expédition de Colomb frappait les imaginations : ce serait alors une pure coïncidence. On ne dispose pas de données précises sur cette situation. Mais on sait que l'esclavage a été une réalité de l’Europe méditerranéenne durant tout le Moyen Âge, l’Espagne étant la plaque tournante de ce trafic aux XIV^e^ et XV^e^ siècles [3]. À Barcelone, au début du XV^e^ siècle, 30 % des esclaves masculins étaient noirs, les femmes noires en représentant 10 %. On retrouve ici l'hypothèse soutenue notamment par Froment selon laquelle l’Afrique serait le berceau de toutes les tréponématoses [19].

Cette version de l'origine de la syphilis est très contestée car la traite négrière est très antérieure aux découvertes portugaises, et parce que les médecins arabes auraient depuis longtemps observé et décrit la syphilis, ce qui n'a pas été le cas.

## Des avancées récentes significatives

Les études précédentes se sont essentiellement fondées sur l'examen de squelettes anciens avant de se tourner vers la phylogénie moléculaire des souches pathogènes qui circulent aujourd'hui, mais aucune de ces approches ne semble décisive : les lésions osseuses ne sont pas toujours typiques et la biologie moléculaire se heurte à la faible variabilité génétique au sein du genre *Treponema*. Une étude des données génétiques de restes humains archéologiques du début de l’ère moderne provenant de Finlande, d’Estonie et des Pays-Bas a permis de reconstruire 4 anciens génomes de *T. pallidum.* Elle a mis en évidence la variété des souches liées à *T. pallidum pallidum* et à la sous-espèce causant le pian. Un génome de haute couverture utilisé pour améliorer les estimations de l'horloge moléculaire place la divergence des sous-espèces modernes de *T. pallidum* fermement à l’époque précolombienne. La distribution spatiale dans la périphérie nord de l’Europe suggère également que les tréponématoses endémiques y étaient largement répandues au début de la période moderne [27, 28].

D'après Schuenemann *et al.*, ces données indiquent que « le pian s'est propagé dans toute l’Europe. Il ne s'est pas limité aux tropiques, comme c'est le cas aujourd'hui ». Les auteurs précisent par ailleurs que « la *datation placerait les premières contagions de T. pallidum pallidum en Europe avant le contact avec le Nouveau Monde, suggérant que l'agent causal originel de l’épidémie continentale à la fin du XV^e^ siècle pourrait avoir résidé dans l’Ancien Monde* » et que donc « *il semble que la première poussée de syphilis connue ne puisse pas être uniquement attribuée aux voyages de Colomb en Amérique* » [35].

Concernant l'hypothèse colombienne, les études archéologiques menées indépendamment par différents chercheurs en 1994 (Powell, Cook, Molto, cités par Harper *et al.*, 2011), tendent à démontrer qu'avant l'arrivée des Européens sur le continent américain, la syphilis n'existait pas en tant que telle, mais qu'il y avait bien des formes de tréponématoses endémiques à transmission non sexuelle. L'archéologie montre en effet chez les Amérindiens -et les ossements disponibles sont très nombreux- de fréquentes lésions tréponé-miques depuis les périodes précolombiennes. Ces squelettes donnent des indices bien plus abondants que dans l’Europe ancienne. Postérieurement aux travaux précédents, ceux de Rothschild *et al.* portant sur 536 restes squelettiques provenant de différents sites de la République dominicaine ont montré 6 % à 14 % de stigmates d'infection tréponémique, émettant l'hypothèse que ce site a été le point de contact initial de la syphilis et de sa propagation ultérieure à l’Ancien monde [23]. Skinner (1994) et Powell (1991,1994) complètent les travaux précédents en estimant qu'il y a bien eu des tréponématoses anciennes en Amérique précolombienne mais que les témoignages de formes congénitales propres à la forme vénérienne de la maladie, en particulier à la Barbade, sont tous postérieurs au XVI^e^ siècle. La syphilis vénérienne serait donc apparue en Amérique après la conquête [26].

Harper *et al*. ont publié une analyse phylogénétique complète de 26 souches géographiquement dispersées de tréponèmes pathogènes. Ce travail a montré que le pian était une maladie ancienne, contrairement à la syphilis, et que de toutes les souches non vénériennes étudiées, les souches les plus proches de celles responsables de l’épidémie de 1495 étaient celles du pian en Amérique du Sud. Cette étude suggère donc que la syphilis, ou un ancêtre de la bactérie, provenait bien du Nouveau Monde [23]. Ces résultats appuient la théorie colombienne de l'origine de la syphilis tout en suggérant que la sous-espèce non sexuellement transmissible est apparue plus tôt dans l’Ancien Monde. Mais ils montrent aussi *a contrario* que la syphilis n'existait pas *stricto sensu* en Amérique à l’époque de Colomb [21, 22]. Cette hypothèse avait déjà été évoquée dans une étude précédente mettant en avant l'absence de preuves solides soutenant l'origine de la maladie dans l’Ancien monde [4].

Pour Arora *et al.*, un travail portant sur des comparaisons phylogénétiques indique que les souches de *T. pallidum pallidum* examinées partagent un ancêtre commun après le XV^e^ siècle, au début de l’ère moderne [5].

Ces différentes études laissent à penser que la syphilis préexistait en Europe avant le retour de Christophe Colomb, peut-être pas sous ses modalités actuelles, ce qui pourrait expliquer qu'elle n'ait pas été clairement perçue par la population ni visible dans les écrits disponibles. Elles ne vont pas non plus dans le sens de l'existence de la syphilis en Amérique à l’époque colombienne. Mais des interrogations subsistent, montrant qu'il est possible que les compagnons de Christophe Colomb aient rapporté en Europe une souche de tréponématose. Une mutation par adaptation aux conditions environnementales aurait alors renforcé son pouvoir pathogène et, lors de son transfert sur de nouveaux sujets - vraisemblablement des prostituées - aurait modifié son mode de transmission. En retour, cette souche aurait contaminé le continent américain par le biais des conquêtes espagnoles et/ou peut être le transport d'esclaves noirs.

La syphilis vénérienne est certainement la maladie infectieuse dont l'origine est la plus débattue, partisans et adversaires d'une provenance américaine continuant de s'affronter au gré des découvertes issues de l'anthropologie du squelette et de la biologie moléculaire.

En fait, la controverse semble dépassée grâce aux travaux plus récents. Le chevauchement significatif des manifestations cliniques parmi les tréponématoses suggère que la question d'origine la plus pertinente ne concerne pas l'origine d'une maladie - à savoir si la syphilis ou le pian était présent en Europe avant 1492 - mais l’évolution et les distributions géographiques de la sous-espèce *T. pallidum pallidum.* Une hypothèse unitaire s'est faite jour, qui postule qu’à partir d'une espèce unique, les sous-espèces phylogénétiques affectant l'homme *(pallidum, pertenue, endemicum)* seraient liées à une différenciation qui s'est sans doute déroulée sur une très longue période, pluri millénaire, sans être intrinsèquement liées à des maladies contemporaines individuelles (syphilis, pian, béjel) [16]. Les phénotypes contemporains de ces maladies pourraient être dus à l'environnement, à l’épidémiologie (comme la voie de transmission) ainsi qu’à des facteurs liés à l'hôte [8, 12, 16, 19, 21]. Crosby suggère d'ailleurs que « les différentes conditions écologiques ont produit différents types de tréponématoses et, avec le temps, des maladies étroitement liées mais différentes » [12].

Alors, est-il possible que l’épidémie de « syphilis » à la fin du XV^e^ siècle en Europe soit due à *T. pallidum pertenue*, en se référant au prix Nobel Charles Nicolle qui considérait les maladies infectieuses comme des espèces vivantes qui naissent, meurent et se transforment ?

Au total, à ce stade, aucune étude ne rassemble de façon satisfaisante l'ensemble des arguments ni n'apporte les preuves réellement décisives en faveur de l'une ou l'autre théorie.

Quelle que soit l'origine retenue, la vision prémonitoire en 1550, de Jean Fernel, premier médecin du roi Henri II, reste d'actualité :

*Ce mal, à moins qu'un Dieu tout puissant, dans sa clémence, ne l'extirpe lui-même, ou que la luxure effrénée des hommes diminue, je crois qu'il sera toujours le compagnon du genre humain*.

## Contribution des auteurs

Jean-Paul Louis : Conception de l’étude, recherche bibliographique, rédacteur principal du manuscrit.

Francis Louis : Lecture et correction du manuscrit.

## Déclaration d'intérêt

Les auteurs ne rapportent aucun conflit d'intérêt. Ils sont seuls responsables du contenu et de la rédaction de l'article.
