# Supplementary material for: Origin of Syphilis in Europe: End of a Controversy?
Source: Med Trop Sante Int. 2025 Mar 21;5(1):mtsi.v5i1.2025.666. [Article in French] doi: 10.48327/mtsi.v5i1.2025.666 (PMC12001989; doi:10.48327/mtsi.v5i1.2025.666)
Supplement: Related texts

# Research article: Origin of Syphilis in Europe: End of a Controversy?

The commonly accepted theory holds that the presence of syphilis in Europe, presumed to be endemic in the Americas, is linked to its introduction by Christopher Columbus. This Columbian origin enjoys broad consensus, which persists in light of recent publications by recognized experts: *“During the Renaissance, syphilis was brought back by Christopher Columbus from America, where it was endemic. This disease, previously unknown and often rapidly fatal, had not been described by Greek physicians, who were considered infallible.”* [9].

Conversely, paleopathological research conducted in recent decades points toward an ancient, pre-Columbian origin of syphilis in Europe, leading some scholars to adopt a more cautious stance. As Bueno notes: *“While smallpox, which spread at record speed in the New World, came from Europe, it seems that syphilis was imported from the Americas.”* [11].

The origins of syphilis remain a subject of passionate debate. The evolutionary history of this sexually transmitted disease is generally framed by two opposing theories: the “Americanists,” who argue that the disease was introduced to Europe by Columbus's crew returning from the New World in 1493, and those who support the idea that the infection was already present in Europe and other regions of the Old World before this pivotal date. The intensity of these controversies is partly due to the complexity of the subject, which fuels extensive epidemiological, historical, clinical, bacteriological, and paleopathological discussions [16]. Ultimately, the debate intertwines memory and history, as reflected in Fabiani-Salmon's remark: *“What if the history of medicine could also blur the line between the historical and the memorial?”* [17].

This debate draws upon clinical, epidemiological, bacteriological, and paleopathological evidence [16].

From a clinical perspective, in addition to syphilis, there are three other human treponematoses, each characterized by its mode of transmission and geographic distribution (yaws, bejel and pinta). Venereal syphilis is primarily transmitted sexually among adults but can also be passed congenitally, affecting newborns. It is currently present on all continents. Yaws is an infection found in warm, humid tropical regions and spreads through skin-to-skin contact. Bejel is a treponematosis prevalent in desert and semi-desert areas, transmitted orally during childhood. Lastly, pinta, which spreads via skin contact, is exclusively a dermatological condition found only in parts of Latin America and is therefore not relevant to this discussion. Except for pinta, the three other treponematoses follow a classic but not always systematic progression through three stages:

- Primary stage: This occurs after infection and is marked by a highly contagious inoculation chancre, whose location depends on the mode of transmission, genital for syphilis, cutaneous for yaws, and oral for bejel.
- Secondary stage: Appearing within a few months, this phase is characterized by a wide range of skin manifestations and the development of painless mucosal plaques. Lesions on the palms of the hands and soles of the feet are particularly suggestive.
- Tertiary stage: Developing over years after infection, this stage affects visceral and mucocutaneous tissues. It is at this point that characteristic bone lesions become evident, which are of particular interest to paleopa-thologists. The hallmark finding is gummatous osteoperiostitis, primarily affecting two key sites: the skull and the tibias.

From a bacteriological perspective, the key question is whether there is one or multiple species of *Treponema*. This question remained unresolved for a long time due to the inability to culture *Treponema* bacteria. However, genetic analysis has provided some initial answers.

The lack of bacteriological differences (morphological, biochemical, serological, and immunological) and the extremely small genetic distance between strains identified in patients with syphilis, yaws and bejel, led to the hypothesis that a single species, *Treponema pallidum*, is responsible for a single disease. The different clinical manifestations were thought to result from variations in transmission modes, which, in turn, depended on climate and cultural practices [7].

A more recent view maintains subspecies within the *Treponema pallidum* spp. species: *pallidum* for syphilis (TPA), *pertenue* for yaws (TPE) and *endemicum* for bejel (TEN). Advances in genome sequencing have confirmed this taxonomic view for the three subspecies *pallidum, pertenue* and *endemicum.* However, these minimal genetic differences are not sufficient to distinguish them as species. The significant epidemiological and clinical differences could be related to variations in genes involved in infectivity and virulence, which could explain the changes in mode of transmission, including epidemic and clinical manifestations [36].

Treponematoses cause bone damage in the late stages of the disease, but in congenital syphilis, this damage appears earlier in newborns and children. The osteoperiostitis that selectively affects the skull and long bones forms the basis of paleopathological criteria for identifying treponematoses in skeletal remains from archaeological sites. In congenital forms, which occur exclusively due to venereal transmission, several lesions are highly suggestive: osteochondritis, osteomyelitis, periostitis, dactylitis, and dental abnormalities. Few scholars dispute the validity of these findings in paleopathology.

Thus, hypotheses regarding the emergence and development of syphilis must be evaluated in light of these various elements.

## The Origins of the Story: Christopher Columbus

Upon Christopher Columbus's return, an unknown disease was reportedly detected in Barcelona in 1493 before manifesting in Naples, Italy, in 1494/1495 during a French invasion. Initially observed among the troops, it quickly spread throughout the Italian peninsula, then to France as armies returned home, and eventually across all of Europe. Is there a causal link between Columbus's return to Spain and the outbreak, or is it merely a coincidence? What arguments can be put forward to address this question? Equipped with a carrack, *Santa Maria*, which he commanded himself, and two caravels, *Niña* and *Pinta*, led by Vicente Yáñez Pinzón and Martín Alonso Pinzón respectively, Columbus set sail on August 3, 1492, with 87 sailors of Spanish and other nationalities. On October 12, 1492, he landed on the island of Guanahani in the Bahamas, which he named San Salvador. The indigenous Taíno people welcomed them warmly, and the great sexual freedom of the women, combined with the sailors’ prolonged abstinence, led to numerous sexual encounters. In his logbook (1492-1493), Columbus alludes to these interactions, including those involving Martin Pinzón, with the young Taino girls. He dropped anchor in Cuba on October 28 and arrived in Hispaniola (Santo Domingo) on December 6. In a report to the King of Spain in 1526, the historian Gonzalo Fernandez d’Oviedo wrote that there was an intense “trade” between sailors and Caribbean women in Hispaniola.

When the *Santa Maria* sank, Christopher Columbus was forced to leave part of his crew behind. He returned from the “Indies” with the 2 caravels, 44 sailors, and 6 natives, arriving in Portugal on March 4, 1493. He then landed in Seville on March 31 before traveling to Barcelona on May 7, where he presented the natives he had brought back to the king. He remained there until September 25, 1493, when he embarked on his second voyage with 17 ships and 1,500 men to establish a colony. He returned with only three surviving natives. Columbus would not return to Spain until June 1496. However, it is worth noting that on February 12, 1494, a fleet of 12 ships, under the command of Antonio de Torres, returned to Spain. These ships carried, among other things, a number of sick people - although no specific details were given - as well as 30 Caribbean slaves. In an accompanying letter to his sovereign, Columbus described them as “cannibals, men, women, boys, and young girls” [15].

## The Italian war and the late 15^th^ century

In September 1494, King Charles VIII of France invaded Italy with an army of 15,000 to 20,000 men, including Flemish, Gascon, Swiss, Italian, and Spanish mercenaries. As was customary at the time, they were accompanied by a horde of beggars and prostitutes [33]. The French king conquered Florence, then Rome - abandoned by Spanish and Neapolitan forces- and finally Naples on January 28, 1495. At the time, Naples was under the rule of the King of Aragon, who maintained a garrison of around a thousand soldiers, including Spaniards, Italians, and Germans [18]. By March 1495, a coalition had formed, consisting of numerous Italian, Spanish, and Austrian troops. Facing a growing number of enemies and dealing with the scandalous conduct of his mercenaries - whose debauchery and disorderly behavior had provoked popular hostility - Charles VIII abandoned Naples. He retreated to Fornovo on July 5, 1495, where he was defeated. By this point, the French troops had already been afflicted by a mysterious new disease, described in precise and evocative terms by Marcellus Cumanus, a surgeon in the Venetian army in 1495. He recounted: *“There were many soldiers and infantrymen whom I witnessed suffering from an eruption of fluids, itching, and multiple pustules on their faces and all over their bodies. The disease usually began on the foreskin or just outside of it, appearing as a grain of millet or the size of a chestnut.”* [13] Another Venetian physician, Benedetto, who was present at Fornovo, reported: *“Through venereal contact, a new disease - or at least one unknown to the doctors who preceded us - the French disease—has spread from the West to us.”* [18]

The syphilis epidemic spread rapidly among both sides. As King Charles VIII's mercenaries were forced into demobilization, they dispersed across Europe, carrying the disease with them.

## The Columbian Hypothesis

Some believe that Spanish soldiers serving on both sides were the primary agents in spreading syphilis across Europe. However, this does not explain how they themselves contracted the disease [18]. It seems unlikely that the small contingent returning from Hispaniola could have triggered a widespread epidemic in such a short time, especially since Columbus never mentioned such an illness among his crew. Moreover, he did not hesitate to present the Indigenous people he had brought back to the royal court.

The first person to propose the theory of an imported infection was Ruy Diaz de Isla, a Portuguese surgeon living in Barcelona. In 1539, in his *Treatise Against the Serpentine Disease Commonly Called Bubas*, he wrote:

*I have great experience in this matter because I treated individuals who were part of the first fleet that discovered this land, and among them, many returned afflicted with this disease. I also treated patients suffering from this illness in Barcelona before King Charles entered Naples.*

Diaz de Isla provided the first clinical description of what appeared to be syphilis based on the “numerous cases” he treated in Barcelona, especially his observations of Martin Pinzón who is thus considered the first known recorded case. However, there is an issue with this claim: Martin Pinzón and his crew arrived in Palos de la Frontera on March 15, 1493, far from Barcelona. He remained there due to illness and died quickly, on March 31 of the same year. Nonetheless, it is possible that syphilis at that time could have led to death within a few months [19]. Additionally, Pinzón's two brothers, Vicente and Francisco, who also traveled on the first expedition, continued sailing for two more decades without any recorded health issues. This raises questions about the claim of “numerous sick individuals from the first fleet,” considering how small their numbers actually were.

In 1503, in Hispaniola, Bartolomé de Las Casas began writing his *General History of the Indies*, in which he stated:

*There was one thing on this island that greatly troubled the Spaniards: the disease of the bubas, which in Italy is called the French disease. It is certain that it came from this island, during Columbus's first return to Castile, either with the first Indians or with some Spaniards who had contracted the bubas… I took care to ask the Indigenous people of this island on multiple occasions whether this disease had existed for a long time, and they answered affirmatively. One fact is clear: every Spaniard who did not remain chaste was afflicted with the bubas, and not a single one escaped it.*

Fra Ramon Pané, who participated in Columbus's second voyage, documented a Taíno myth in his *Account of the Ancient History of the Indians*, which described a man who, after engaging with a woman for his own pleasure, quickly sought a place to wash himself, as he was covered in sores, sores that the Europeans later referred to as the “French disease” [31].

In his *General History of the Indies* (1535), Gonzalo Fernández de Oviedo, who was at the Spanish court when Columbus presented the first Indigenous people, wrote:

*Your Majesty can be certain that this disease comes from the Indies, where it is very common among the Indians, though it is not as dangerous there as it is in our lands. The first time this disease was seen in Spain was after the Admiral discovered the Indies and returned from those lands. And when, in 1495, Captain of Cordova traveled to Italy with an army, this disease spread there with some of his Spaniards, and that was the first time it was seen in Italy.*

However, it is worth noting that these Spanish troops arrived in Italy after Charles VIII's forces had already left Naples.

Supporters of the Columbian hypothesis also rely on other historical testimonies from contemporaries, some of whom were direct victims of the venereal disease. One example is the knight Ulrich von Hutten (1488-1523), who dedicated a brief treatise to syphilis in 1521. Other references include Niccolò Leoniceno (1428-1524), Giovanni de Vigo (c. 1450-1525), Girolamo Fracastoro (1478-1553), and Johannes Baptista Montanus (c. 1489-1551). All these texts describe the emergence of an epidemic across Europe between the late 15^th^ and early 16^th^ centuries - a previously unknown disease that deeply impacted society due to its severity and apparent novelty [16].

## The Pre-Columbian Hypothesis: Syphilis and Europe

This hypothesis argues that syphilis existed in Europe before Europeans arrived in the Americas but had not been identified as such. Written records are scarce, as diagnosing the disease is difficult based on descriptions found in ancient and medieval texts predating the 15^th^ century. According to Hippocratic tradition, any skin condition was broadly referred to as *lepra.* It is therefore unsurprising that chroniclers of the time did not provide precise accounts of a disease that had neither been described nor named. It may also have been less virulent, making it rarer and more difficult to distinguish from other conditions with similar clinical manifestations.

Supporters of the pre-Columbian model highlight earlier texts describing a venereal disease in Europe, characterized by genital ulcerations. When accompanied by ulcers on the face and lower limbs, it was sometimes mistaken for leprosy. Bartholomew of Glanville (?-c. 1360) mentioned a congenital and venereal form of leprosy. Bernard of Gordon (13th–14th centuries), Guy de Chauliac (c. 1300–1368), and Valescus of Tarenta (14^th^–15^th^ centuries) referred to penile ulcers occurring after sexual relations with infected women. However, these historical testimonies have been criticized as atypical, unreliable, or inaccurately dated [32]. One debated text is *Sylva in Scabiem (On Ulceration)*, a poem by Angelo Poliziano written between 1475 and 1478, which some suggest describes syphilis, though this interpretation is not fully supported by Moulin and Delort [29]. According to them, *“This case raises the question of syphilis origins without resolving it.”* Paleopathology offers potentially more scientific evidence than disputed textual records. The pioneering work of Hackett and Steinbock in 1976, followed by Resnick in 1988, demonstrated that a detailed morphological study of skeletal lesions could help diagnose treponematoses (the group of diseases that includes syphilis) based on osteo-periostic markers. These studies were expanded in 1995 by Rothschild *et al.*, who developed a method for distinguishing between different types of treponematoses [34]. However, skepticism remains, as distinguishing lesions attributed to treponematoses from skeletal analysis alone is challenging, if not impossible. The main differences between these diseases are primarily seen in the frequency and location of bone involvement [7, 8]. Building on this research, Brun *et al.* conducted an inventory of archaeological and paleopathological evidence of treponematoses predating the 16^th^ century [10]. Their findings suggest that syphilis was already present in Antiquity in England, France, Italy, Austria, and Croatia. A particularly notable case is that of the *Costebelle fetus* (Var, France), dated to the 4th century, which exhibited an exceptional set of bone lesions strongly indicative of congenital syphilis. Other potential cases of congenital syphilis have been identified in various European countries, with subsequent studies further refining these initial observations [2, 3, 14, 20, 24, 30].

Although archaeological evidence remains rare, systematic research - now unencumbered by the long-dominant Columbian hypothesis - may expand our understanding of the history of treponemal infections in Europe. While doubts persist about the reliability of some diagnoses, congenital syphilis cases are considered more definitively identifiable.

## A Portuguese version of the origin of syphilis

In 1991, Livingstone, struck by the temporal and geographical correlation between Portuguese exploration along the African coasts and the outbreak of the syphilis epidemic, proposed that the disease may have been introduced to the Iberian Peninsula by Portuguese sailors [1]. The Portuguese reached Cape Verde as early as 1444, the Gold Coast in 1460, the Congo River mouth in 1483, and the Cape of Good Hope in 1487. In 1444, an expedition returned to Portugal with its first cargo of 235 enslaved people, and to accommodate the expansion of the transatlantic slave trade, the *Casa dos Escravos* (House of Slaves) was established in Lisbon in 1486. Cases of *yaws (pian)*, a disease caused by a close relative of the syphilis bacterium, may have been brought back to Europe, where environmental and cultural factors could have altered its characteristics. An epidemic hotspot might have emerged between 1460 and 1490, slowly spreading before erupting at the end of the century, coinciding with the return of Columbus’ expedition, making their connection purely coincidental. No precise data confirm this hypothesis, but historical records establish that slavery was widespread in the Mediterranean throughout the Middle Ages, with Spain serving as a major hub for the trade in the 14^th^ and 15^th^ centuries [3]. In early 15^th^ -century Barcelona, 30% of enslaved men were Black, while Black women accounted for 10% of the enslaved population. This aligns with Froment's hypothesis that Africa was the cradle of all *treponematoses* (the group of diseases including syphilis) [19].

This version of syphilis’ origins remains highly contested, as the slave trade predates Portuguese explorations, and Arabic medical sources, which had long documented various diseases, do not describe syphilis in earlier periods.

## Significant recent advances

Previous studies primarily relied on the examination of ancient skeletons before shifting toward the molecular phylogeny of present-day pathogenic strains. However, neither approach has provided definitive answers: bone lesions are not always distinctive, and molecular biology faces challenges due to the low genetic variability within the *Treponema* genus.

A study analyzing genetic data from early modern human remains found in Finland, Estonia, and the Netherlands successfully reconstructed four ancient *T. pallidum* genomes. It identified the diversity of strains linked to *T. pallidum pallidum* (the causative agent of syphilis) and the subspecies responsible for *yaws*. A high-coverage genome, used to refine molecular clock estimates, placed the divergence of modern *T. pallidum* subspecies firmly in the pre-Columbian era. The geographical distribution of these strains in northern Europe further suggests that endemic *treponematoses* were widespread across the continent at the dawn of the modern era [27, 28].

According to Schuenemann *et al.*, these findings indicate that *yaws* once spread throughout Europe and was not confined to tropical regions as it is today. The authors also state that *“the dating places the first infections of T. pallidum pallidum in Europe before contact with the New World, suggesting that the original causative agent of the continental epidemic at the end of the 15^th^ century may have already existed in the Old World.”* Thus, *“it appears that the first known outbreak of syphilis cannot be solely attributed to Columbus’ voyages to the Americas.”* [35].

Regarding the Columbian Hypothesis, archaeological studies conducted independently by various researchers in 1994 (Powell, Cook, Molto, as cited by Harper *et al.*, 2011) suggest that before Europeans arrived in the Americas, syphilis did not exist as such, but rather that non-sexually transmitted endemic forms of treponematoses were present. Indeed, archaeology reveals frequent treponemal lesions among Indigenous populations - whose skeletal remains are abundant - dating back to pre-Columbian times. These skeletons provide significantly more evidence than those from ancient Europe. Following these studies, Rothschild *et al.* examined 536 skeletal remains from various sites in the Dominican Republic, identifying treponemal infection markers in 6% to 14% of cases. They hypothesized that this site might have been the initial point of contact for syphilis before its subsequent spread to the Old World [23].

Skinner (1994) and Powell (1991, 1994) expanded on previous research, suggesting that while ancient treponematoses existed in pre-Columbian America, evidence of congenital forms characteristic of the venereal disease - especially in Barbados - only appears after the 16^th^ century. This implies that venereal syphilis emerged in the Americas following European conquest [26]. Harper *et al.* conducted a comprehensive phylogenetic analysis of 26 geographically dispersed strains of pathogenic treponemes. Their findings showed that yaws was an ancient disease, unlike syphilis, and that among all non-venereal strains studied, those most closely related to the 1495 epidemic-causing strain originated from yaws in South America. This suggests that syphilis, or an ancestor of its bacterium, indeed came from the New World [23]. These results support the Columbian theory of syphilis origins while also indicating that a non-sexually transmitted subspecies emerged earlier in the Old World. However, they also suggest that syphilis, in the strictest sense, did not exist in the Americas at the time of Columbus [21, 22]. This hypothesis had previously been raised in a study emphasizing the lack of solid evidence supporting the disease's Old World origin [4].

According to Arora *et al.*, phylogenetic comparisons indicate that the examined strains of *T. pallidum pallidum* share a common ancestor dating to the 15^th^ century, at the dawn of the modern era [5].

These various studies suggest that syphilis may have preexisted in Europe before Columbus’ return, though not necessarily in its current form, which could explain why it was not clearly perceived by the population or documented in written records. They also challenge the idea that syphilis existed in the Americas during Columbus’ time. However, uncertainties remain, and it is possible that Columbus’ crew brought back a treponematosis strain that, through environmental adaptation, underwent a mutation that increased its pathogenicity. When transmitted to new individuals - likely prostitutes - this strain could have altered its transmission mode. In turn, this new strain may have recontaminated the Americas through Spanish conquests and/or the transport of enslaved Africans.

Venereal syphilis is arguably the most debated infectious disease in terms of origin, with proponents and opponents of an American provenance continuing to clash as new discoveries emerge from skeletal anthropology and molecular biology. In reality, recent research suggests that the controversy may now be outdated. The significant overlap in clinical manifestations among trepo-nematoses implies that the most relevant question is not whether syphilis or yaws was present in Europe before 1492, but rather the evolution and geographic distribution of the *T. pallidum pallidum* subspecies. A unified hypothesis has emerged, proposing that all phylogenetic subspecies affecting humans *(pallidum, pertenue, endemicum)* originated from a single ancestral species. Their differentiation likely occurred over a millennia-long period, independent of contemporary disease classifications (syphilis, yaws, bejel) [16]. The modern phenotypes of these diseases may result from environmental factors, epidemiological variables (such as transmission routes), and host-related factors [8, 12, 16, 19, 21]. Crosby even suggested that “different ecological conditions produced different types of trepo-nematoses, which, over time, evolved into closely related but distinct diseases” [12].

Could the “syphilis” epidemic of the late 15^th^ century in Europe have been caused by *T. pallidum pertenue*? Nobel Prize-winning microbiologist Charles Nicolle once described infectious diseases as living species that are born, evolve, and transform.

Ultimately, no study has yet gathered all available arguments or provided decisive proof in favor of any one theory.

Regardless of its origin, the prophetic vision of Jean Fernel, chief physician to King Henry II of France, remains relevant even today. In 1550, he wrote:

This evil, *unless an all-powerful God, in His mercy, eradicates it Himself, or the unbridled lust of men diminishes, I believe always be the companion of the mankind*.

## Authors’ contributions

Jean-Paul Louis: Study design, bibliographic research, main author of the manuscript.

Francis Louis: Reading and correction of the manuscript.

## Declaration of interest

The authors have no conflicts of interest to declare. They are solely responsible for the content and writing of the article.
